# Association between serum bilirubin levels and carotid atherosclerosis: a systematic review and meta-analysis

**DOI:** 10.3389/fendo.2025.1451465

**Published:** 2025-06-19

**Authors:** Bao-E Yan, Ying Li, Min-Jie Zhu, Qian-Jun Wang, Jing Xiao, Yan Zhang, Chun-Yan Zhang, Jing Zhou, Tuo Han

**Affiliations:** ^1^ Department of Cardiovascular Medicine, The Second Affiliated Hospital of Xi’an Jiaotong University, Xi’an, Shaanxi, China; ^2^ Department of Cardiovascular Medicine, Affiliated Hospital of Yan’an University, Yan’an, Shaanxi, China

**Keywords:** carotid atherosclerosis, carotid intima-media thickness, carotid plaque, bilirubin, meta-analysis

## Abstract

**Objective:**

Carotid atherosclerosis (CAS) is a significant factor contributing to cardiovascular events and poses a major public health concern. There are still many controversies about the association between serum bilirubin and CAS. This study aims to provide a systematic review and meta-analysis to examine the association between serum bilirubin levels and carotid atherosclerosis.

**Methods:**

An electronic literature search was performed using PubMed, Web of Science and Embase up to December 2023. Articles were screened based on predefined inclusion criteria and assessed for risk of bias and quality of evidence utilizing the Newcastle-Ottawa Scale and GRADE tool. Pooled mean differences were calculated using a random effects model. Subgroup and meta-regression analyses were performed to identify potential sources of heterogeneity.

**Results:**

Nine studies involving 7,023 participants were included in this meta-analysis. The results indicated that patients with carotid atherosclerosis exhibited lower levels of total bilirubin compared to those without (SMD -3.42, 95% CI [-5.18, -1.67]), with a statistically significant difference (z=-3.819, *P*<0.001). Moreover, a significant inverse association was found between total bilirubin levels and the risk of carotid atherosclerosis (OR 0.79, 95% CI [0.71, 0.88], *P*<0.001, I²=78.2%). However, substantial heterogeneity was observed (I²=98.0%, *P*<0.001). Subgroup and meta-regression analyses indicated that sample size and the severity of carotid atherosclerotic lesions might contribute to the heterogeneity observed across studies. The GRADE assessment was low.

**Conclusion:**

Lower serum bilirubin levels are associated with an increased risk of carotid atherosclerosis. This meta-analysis offers new insights into the development of diagnostic biomarkers and therapeutic targets. Further prospective cohort studies are necessary to validate our conclusions.

**Systematic Review Registration:**

https://www.crd.york.ac.uk/PROSPERO/, identifier CRD42023447199.

## 1 Introduction

Cardiovascular disease (CVD) is the leading cause of disability and premature mortality worldwide, imposing a substantial burden on individuals, families, and healthcare systems. According to the Global Burden of Cardiovascular Diseases and Risks Study 1990-2022, the age-standardized mortality rate for CVD has decreased by 34.9%. However, the total number of CVD-related deaths surged from 12.4 million in 1990 to 19.8 million in 2022 (1). This trend underscores the aging global population and the rising prevalence of cardiometabolic risk factors, including high systolic blood pressure, elevated low-density lipoprotein (LDL) cholesterol, increased body mass index (BMI), high fasting glucose, and renal insufficiency (2). Prolonged exposure to these cardiometabolic risk factors predisposes individuals to the formation and development of atherosclerotic plaques, facilitating the transition from subclinical to clinical stages (3).

Atherosclerosis (AS) is a chronic inflammatory disease primarily driven by lipid deposition, typically resulting in the narrowing of the vessel lumen and plaque formation (4). In cases of carotid artery stenosis, carotid intima-media thickness (CIMT) and plaque can be routinely assessed using non-invasive Doppler ultrasonography (5). In the global population aged 30–79 years in 2020, the prevalence of increased carotid intima-media thickness (≥1.0 mm) was estimated at 27.62%, affecting over one billion individuals, while the prevalence of carotid plaque was approximately 21.13%, and carotid stenosis was 1.50% (6).Thus, the considerable disease burden of CAS has prompted efforts toward effective preventive health strategies and early assessment.

Bilirubin, recognized as the terminal product of heme catabolism, has traditionally been viewed as potentially harmful, with elevated levels associated with liver dysfunction and neurological deficits (7, 8). However, emerging evidence indicates that bilirubin possesses significant anti-inflammatory, antioxidant, and neuroprotective properties when acting as a hormone (9, 10). It protects LDL from peroxidation and endothelial cells against oxidative damage, while also enhancing heme oxygenase activity and serum cholesterol solubility (11). Elevated serum bilirubin levels have been positively correlated with total serum antioxidant status and inversely correlated with pro-inflammatory cytokines such as IL-1β, IL-6, and TNF-α, as well as other atherogenic factors, including ICAM, E-selectin, or VEGF in patient with atherosclerosis (12). Furthermore, bilirubin has demonstrated clinical significance in diagnosising and prognosticating oxidative stress-related diseases, including stroke (13), peripheral arterial disease (14), chronic kidney disease (15) and cancer (16), suggesting that regulating bilirubin levels may offer a promising a therapeutic strategy for these conditions.

The relationship between serum bilirubin levels and carotid atherosclerosis has garnered increasing scholarly attention, yielding conflicting results across studies. Consequently, we conducted a meta-analysis to thoroughly investigate the associations between serum bilirubin levels and carotid atherosclerosis, specifically focusing on carotid intima-media thickness (CIMT), atherosclerotic plaque and stenosis. This study may provide a foundation for understanding the implications of serum bilirubin levels in CAS and offer new insights for early diagnosis and potential therapeutic targets.

## 2 Methods

### 2.1 Search strategy

This study was conducted in accordance with the Preferred Reporting Items for Systematic Reviews and Meta-Analyses (PRISMA) guideline (17). A comprehensive electronic literature search was performed to identify eligible articles from PubMed, Web of Science and Embase, covering the period from database inception to December 2023. The search utilized the following terms and keywords: (“Bilirubin”[Mesh] OR “bilirubinaemia” OR “hyperbilirubinemia” AND (“Carotid Artery Diseases”[Mesh]) OR (“Carotid Intima-Media Thickness”[Mesh] OR “Carotid Atherosclerosis” OR “carotid plaque”). The specific search strategy is provided in the **Supplementary Table S1**. The search was not limited by language. The protocol of this meta-analysis has been registered in PROSPERO (CRD42024498887).

### 2.2 Study selection

Inclusion criteria for included studies are as follows: (1) observational studies(cohort, case-control, and cross-sectional studies); (2)the relationship between circulating total bilirubin level and carotid atherosclerosis was investigated; (3) studies reporting mean values with standard errors or odds ratio (OR) with the corresponding 95% confidence intervals (CIs) for the risk of carotid atherosclerosis or other adverse clinical outcomes; and (4) examination of carotid atherosclerosis based on ultrasonography. Exclusion criteria are as follows: (1) the study did not report the relationship between total bilirubin level and carotid atherosclerosis; (2) the study did not provide complete effect estimates or data were not available; (3) articles not published in English, and (4) the study did not involve human subjects. Two investigators (EBY/YL) independently assessed the eligibility of all studies for inclusion. If conflicts arose during the process, a third investigator (TH) adjudicated the contradiction through discussion.

### 2.3 Data extraction and quality assessment

Data extraction was managed in Microsoft Excel. Two investigators (EBY and YL) independently extracted the following information from the included articles, with any disagreements resolved through discussion with a third investigator (TH): (1) Study details, including first author, publication year, country, sample size, and design; (2) Participant characteristics, including number, age, sex, and bilirubin levels; (3) Evaluation/indicator of carotid atherosclerosis.

The methodological quality of each study was assessed independently by two investigators (EBY/YL) using the Newcastle-Ottawa Scale (NOS) (18). Each included study was rated on a scale of 1 to 9 points based on three criteria: (1) study selection criteria; (2) comparability of groups; and (3) evaluation of the outcome/exposure.

### 2.4 Certainty of evidence assessment

The GRADE tool was employed to evaluate the quality of the pooled outcomes derived from the effect estimates (19). The quality of evidence was classified into four categories: “very low,” “low,” “moderate,” and “high.” A classification of “very low” suggests that the true effect may differ substantially from the estimate, indicating a high degree of uncertainty in the results. In contrast, a classification of “low” reflects limited confidence in the effect, while “moderate” implies that the true effect may be close to the estimate, albeit with a possibility of variation. The scores were reviewed by the senior author (TH).

### 2.5 Statistical analyses

In our meta-analysis, we utilized the standardized mean difference (SMD) and pooled odds ratios (ORs) to examine the association between serum bilirubin and carotid atherosclerosis. We assessed heterogeneity among the various studies using the I^2^ test and Cochran’s Q-test (20). A fixed-effects model was applied for low heterogeneity (I²<50%), while a random-effects model was employed for high heterogeneity (I²>50%). Subgroup analyses were conducted based on study variables such as design, average age, sample size, country, and year of publication. To investigate the sources of heterogeneity, we performed univariate meta-regression analysis. Additionally, sensitivity analysis was executed by excluding each study to evaluate the robustness of the results (21). Publication bias was assessed through funnel plot, with Egger’s test and Begg’s test utilized for visualization (22). A two-sided *P* value of less than 0.05 was considered statistically significant. To maintain unit consistency across all included studies, we converted serum bilirubin levels from mg/dL to μmol/L by multiplying by 17.1. Statistical analyses were performed using Stata (version 18.0).

## 3 Results

### 3.1 Literature search and study characteristics

**Figure 1:**
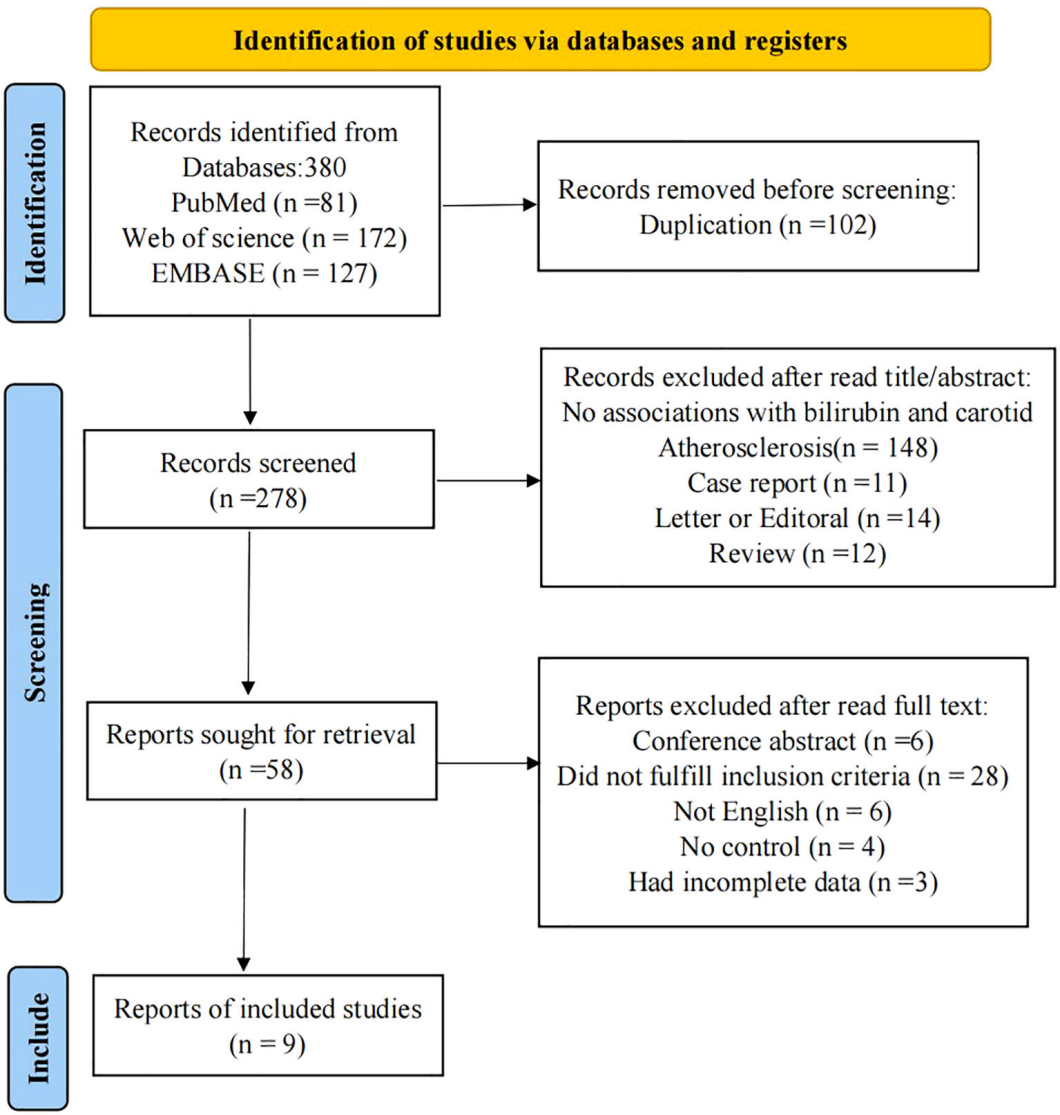
PRISMA Flow chart of the study collection for the present review and meta-analysis.

We identified a total of 380 potential studies across six electronic databases: PubMed(81), Web of Science(172), and EMBASE(127), utilizing a comprehensive search strategy. Automation tools facilitated the exclusion of 102 articles. Subsequently, after a thorough review of titles and abstracts, we excluded 220 studies, which comprised 148 studies that did not report the relationship between serum bilirubin and carotid atherosclerosis, 12 reviews, 14 letters, editorials and commentaries, 11 case reports, and 35 studies focused on animal models, chemistry, or cell lines. Following a full-text review, we excluded an additional 47 studies: 6 studies were removed due to being non-English, 4 lacked a control group, and 28 were considered irrelevant to the topic and did not meet our inclusion criteria. During data extraction phase, we found that 3 studies lacked sufficient information. Consequently, a total of 9 studies were ultimately included in our meta-analysis (23–31). The flow chart illustrating the screening process is shown in **Figure 1**.

### 3.2 Characteristics of included studies

The included studies comprised two longitudinal study and seven cross-sectional studies, yielding a total of 9 articles for this meta-analysis. The combined sample size was 9,398 participants, with 5,708 individuals in the carotid atherosclerosis group and 3,690 in the control group. The study with the largest sample size included 4360 subjects (Lu Qin, 2023, Shijiazhuang, China), while the smallest included 80 participants (JIANG Shi-min, 2012, Beijing, China). Participant ages ranged from 40 to 78 years. Besides healthy middle-aged and elderly individuals, the study population included individuals with prehypertension, essential hypertension, HIV-1 infection, obstructive sleep apnea, prediabetes, and hyperbilirubinemia. Five studies defined subclinical carotid atherosclerosis or carotid atherosclerosis as a carotid intima-media thickness (CIMT) of greater than or equal to 0.9 mm (24, 26–28, 30). Two studies defined a carotid atherosclerotic plaque based on a CIMT of greater than or equal to 1.2 mm or the presence of wall irregularities with thickening or a convex luminal localization (23, 25). The remaining studies established carotid atherosclerotic plaque based on a CIMT of greater than or equal to 1.5 mm or focal thickening exceeding 50% (29, 31). The basic characteristics of the literature are shown in **Table 1**.

**Table 1:** The characteristics of the included studies in the meta-analysis.

Author	Year	Country	Study Design	Study Population	Carotid Atherosclerosis				Controls				Definition of Carotid Atherosclerosis	Effect value
					Age, years (mean, SD)	Male, %	n	TBIL (mean ± SD, umol/L)	Age, years (mean, SD)	Male, %	n	TBIL (mean ± SD, umol/L)		
Zhao J (25)	2019	China	Cross-sectional	Hospital-based;essential hypertension;patients(n=318)	65.21 ± 6.43	99(50.77)	195	11.78 ± 2.89	65.21 ± 6.43	58(47.15)	123	14.31 ± 3.74	Thickening of the irregular wall of the tube wall, IMT≥1.2 cm and the local structural changes of the convex lumen, are regarded as indicating the formation of atherosclerotic plaques.	OR:0.71(0.54,0.92)
Tang L H (30)	2019	China	Cross-sectional	prehypertensive patients from a community in Guangzhou	52.69 ± 11.58	50(49.50)	101	8.50 ± 4.17	50.28 ± 10.33	332(56.27)	590	15.13 ± 6.57	A CIMT< 0.9 mm was defined as normal CIMT, subclinical atherosclerosis was defined as CIMT 0.9–1.2 mm, whereas atherosclerosis was defined as CIMT ≥ 1.2 mm according to previously described.	OR:0.476(0.253,0.764)
Muccini C (29)	2018	Italy	Cross-sectional	Hospital-based;adult HIV-1–infected	54.4 (50.1–60.5)	420 (82.2)	511	46.85(29.17–88.4)	49.9 (44.9–54.2)	313 (79.8%)	392	49.50 (29.17–106.08)	The morphological examination was performed bilaterally at common carotid (CCA) and bifurcation (BIF), reporting the highest values of IMT retained in each patient. The presence of carotid lesions was defined as an IMT ≥1.5 mm in ≥1 region at carotid ultrasound. The stenosis was graded according to the European Carotid Surgery Trial (ECST).	OR:0.57 (0.36 to 0.90)
Lyu Q S (24)	2018	China	Cross-sectional	Hospital-based;prehypertension patients(n=219)	53.38 ± 9.22	25(56.82)	44	9.43 ± 3.27	52.91 ± 10.16	93(53.14)	175	14.38 ± 4.49	A CIMT <0.9 mm was defined as normal CIMT, CIMT ≥0.9 mm was increased CIMT, namely subclinical atherosclerosis according to the 2007 Guidelines of the European Society of Cardiology (ESC)	OR:0.483(0.240,0.972)
Duman H (26)	2018	Turkey	Cross-sectional	Hospital-based; Obstructive sleep apnea (OSA)patients (n=84)	49 ± 6.8	26 (60.5)	43	8 ± 3.4	50 ± 8.2	23 (56.1)	41	12.3 ± 4.1	Patients were divided into two groups according to whether the CIMT measurement was less than 0.9 mm. The mean CIMT was calculated based on the average of three measurements from both carotid arteries.	OR:0.72 (0.60–0.86)
Hamur H (28)	2016	Turkey	Cross-sectional	Hospital-based;Prediabetes patients(n=170)	53.8 ± 7.3	49 (65.3)	75	7.5 ± 3.8	54.6 + 7.8	58 (61.1)	95	11.4 ± 4.8	Prediabetic patients with CIMT < 0.9 mm were accepted as group 1, and the remaining participants (CIMT ≥ 0.9 mm) were acknowledged as group 2	OR:0.866 (0.800-0.937)
Jiang S M (23)	2012	China	Prospective cohort	healthy population;recruited from 10 communities(n=780)	67.6 ± 10.2	44 (55.0)	51	14.4 ± 5.9	60.7 ± 10.4	44 (55.0)	29	17.5 ± 7.0	Carotid atherosclerotic plaques were defined as localized echogenic lesions with carotid intima-media thickness(CIMT) ≥1.2 mm	HR:0.915 (0.844-0.992)
Yang X F (27)	2009	China	Cross-sectional	Hospital-based;primary hypertension(n=198)	65.7 ± 7.1	104 (52.6)	133	12.8 ± 1.3	64.5 ± 6.2	34 (52.3)	65	16.8 ± 1.5	Plaque was defined as clearly isolated focal thickening of the intimal-medial layer with an intimal-medial thickness of ≥1.3 mm at the common or internal carotid arteries or the carotid bulb. Carotid intima-media wall thickening was said to occur when the intima-media thickness, which was measured at the far wall of the distal 10 mm of the common carotid artery, was ≥0.9 mm. The presence of intima-media wall thickness or plaque was regarded as a sign of atherosclerosis of the carotid artery.	OR:0.575 (0.370-0.755)
Qin L (31)	2023	China	Retrospective cohort	included 4360 subjects who underwent health examinations regularly in Hebei General Hospital	49.7 ± 10.4	1428 (65.0)	2180	14.4 ± 4.5	49.3 ± 10.0	1420 (65.1)	2180	14.5 ± 4.6	The presence of a carotid plaque was defined as the focal abnormal wall thickness (IMT >1.5 mm) or focal thickening of the surrounding IMT >50%.	HR:0.963(0.922-1.005)

### 3.3 Quality analysis

Quality evaluation indicated a clear diagnosis of carotid atherosclerosis, appropriate methods for detecting total bilirubin levels, satisfactory group comparisons, and complete data (**Supplementary Table S2**). According to the Newcastle-Ottawa Scale (NOS) guidelines, all included studies achieved a total score of 8 stars or higher, signifying good overall study quality.

### 3.4 Meta-analysis results

**Figure 2:**
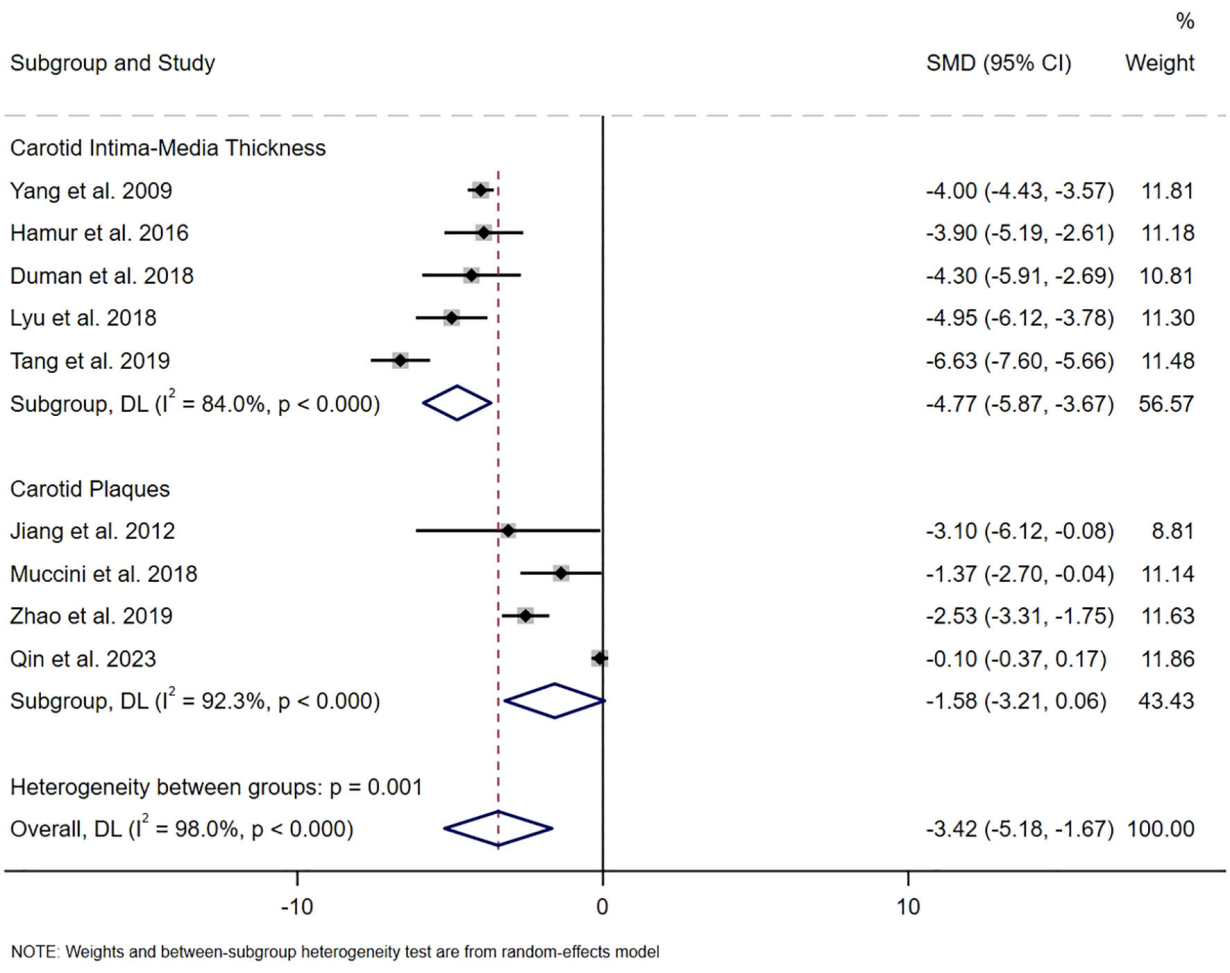
The SMD on the relationship between serum bilirubin level and the risk of carotid atherosclerosis.

The distribution of studies estimating the association between the serum bilirubin level and carotid atherosclerosis is illustrated in **Figure 2**. A total of nine studies had calculated the pooled SMD, revealing lower serum bilirubin levels in patients with carotid atherosclerosis compared to those without (SMD -3.42, 95% CI [-5.18, -1.67]), with a statistically significant difference (z=-3.819, *P*<0.001). Due to significant heterogeneity (I^2^ = 98.0%, *P*<0.001), a random-effects model was employed. Subsequently, analyses were conducted for carotid plaques and intima-media thickness, respectively. For intima-media thickness, five studies involving 1,362 individuals were included. Regarding the presence of plaques, four observational studies with 5,661 individuals were analyzed. We found that total bilirubin levels were negatively associated with carotid intima-media thickness (SMD -4.77, 95% CI [-5.87, -3.67], *P*< 0.001, I^2^ = 84.0%), but not with carotid plaques (SMD -1.58, 95% CI [-3.21, 0.06], *P*=0.058, I^2^ = 92.3%), as shown in **Figure 2**.

**Figure 3:**
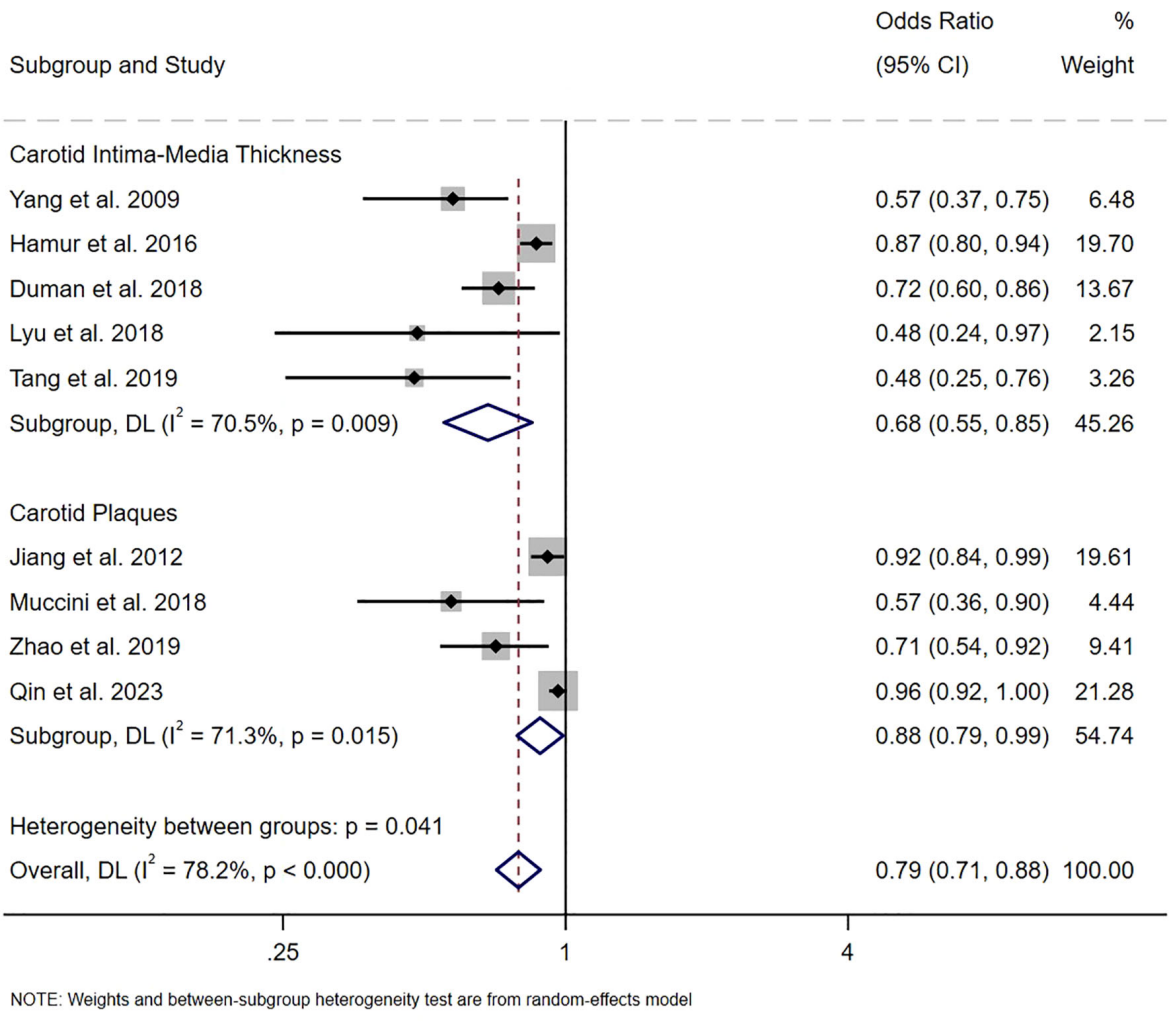
The pooled ORs on the relationship between serum bilirubin level and the risk of carotid atherosclerosis.

Nine studies calculated the pooled ORs, demonstrating a significant negative relationship between serum bilirubin levels and the risk of carotid atherosclerosis (OR 0.79, 95% CI [0.71, 0.88], *P*<0.001, I^2^ = 78.2%, **Figure 3**). Pooled data suggested that the serum bilirubin levels were associated with a 0.68-fold decrease in the odds of having a higher carotid intima-media thickness (OR 0.68, 95% CI [0.55, 0.85], P<0.001, I^2^ = 70.5%, GRADE: low). The pooled analysis indicated that serum bilirubin levels serve as a protective factor against an increased risk of carotid plaque (OR 0.88, 95% CI [0.79, 0.99], P<0.001, I^2^ = 71.3%, GRADE: low), indicating a negative correlation between serum bilirubin levels and the occurrence of carotid atherosclerosis. The GRADE evidence ratings are specified in **Supplementary Table S3**.

### 3.5 Subgroup analysis

**Figure 4:**
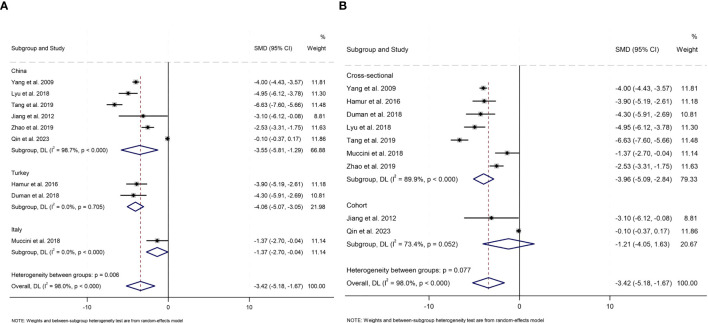
Subgroup analysis of meta-analysis **(A)** Country **(B)** Study design.

To investigate the impact of study design, geographical ethnicity, publication year, sample size, and average age and diagnostic criteria for carotid atherosclerosis on serum bilirubin levels in carotid atherosclerosis patients and controls, prespecified subgroup analyses were performed (**Figure 4**, **Supplementary Figure S1**).

All studies were categorized into three subgroups (China, Turkey and Italy) based on the countries were conducted. Significant differences in serum total bilirubin levels between carotid atherosclerosis patients and controls were observed in subgroups from China (SMD -3.55, 95% CI [-5.81, -1.29], *P*= 0.002), Turkey (SMD -4.06, 95% CI [-5.07, -3.05], *P*<0.001), and Italy (SMD -1.37, 95% CI [-2.70, -0.04], *P*= 0.043). Notably, the heterogeneity in the China group remained high (I^2^ = 98.7%), whereas the Turkey and Italy groups exhibited a substantial decrease in heterogeneity (I^2^ = 0.0%). Subgroup analysis based on study design revealed that, among cross-sectional studies, patients with carotid atherosclerosis exhibited significantly lower serum bilirubin levels compared to controls (SMD -3.96, 95% CI [-5.09, -2.84], *P*<0.001). In contrast, among cohort studies, no significant difference in serum bilirubin levels was observed between patients with carotid atherosclerosis and controls (SMD -1.21, 95% CI [-4.05, 1.63], *P*=0.404); however, high heterogeneity persisted in both groups (I^2^ = 89.9%; I^2^ = 73.4%). Lower levels of serum bilirubin were observed among patients with carotid atherosclerosis across both subgroups stratified by sample size. In the subgroup analysis involving fewer than 200 participants, the SMD was -3.99 (95% CI [-4.38, -3.60], *P*<0.001) with an I^2^ of 0.0%. Similarly, in the subgroup analysis of 200 or more participants, the SMD was -3.10 (95% CI [-5.71, -0.50], *P* = 0.020), with an I^2^ of 98.3%. As for age, a significant difference in serum bilirubin levels was observed in individuals aged younger than 60 years (SMD -3.53, 95% CI [-6.21, -0.84], *P*= 0.010, I^2^ = 98.0%), as well as in those aged 60 years and older (SMD -3.28, 95% CI [-4.53, -2.03], *P*<0.001, I^2^ = 81.4%). When analyzing the data by publication year, patients with carotid atherosclerosis consistently exhibited lower serum bilirubin levels compared to controls. Specifically, studies published before 2016 reported an SMD of -3.97 (95% CI [-4.38, -3.57], *P*<0.001, I^2^ = 0.0%), while studies published after 2017 showed an SMD of -3.29 (95% CI [-5.66, -0.93], *P*=0.006, I^2^ = 98.0%). Subgroup analysis was analyzed according to the diagnostic criteria for carotid atherosclerosis, and when CIMT ≥ 0.9 mm was used as a diagnostic criterion, serum bilirubin levels were significantly lower in carotid atherosclerosis patients than in controls (SMD=-4.77, 95% CI: [-5.87, -3.67], *P*<0.001, I²=84%). However, in studies where the criteria were CIMT ≥ 1.2 mm or plaque formation, or CIMT ≥ 1.5 mm or focal thickening > 50%, no significant difference in serum bilirubin levels was found between patients and controls (*P* > 0.05), with reduced heterogeneity (I²=0.0%, I²=70.4%). The subgroup analysis indicated a significant difference in serum bilirubin levels between the carotid atherosclerosis group and the control group.

### 3.6 Meta-regression

Due to significant heterogeneity (I^2^ = 98.0%) observed in the meta-analysis, a univariate meta-regression analysis was conducted to identify potential sources of this heterogeneity (**Table 2**, **Supplementary Figure S2**). The results of meta-regression analysis, which examined factors such as on country, study design, research sample size, average age, and publication year and male ratio of carotid atherosclerosis groups, indicated that none of them were potential sources of heterogeneity (all *P >*0.05). However, sample size and the severity of carotid atherosclerosis lesions(CIMT or carotid plaques) demonstrated significant effects on the meta-analysis outcomes (*P* < 0.05), suggesting they may be sources of heterogeneity.

**Table 2:** Results of Meta-Regression analysis.

Variable	Coefficient	Standard error	95% CI	t	*P*
Country	0.6572105	1.041576	(-1.805725, 3.120146)	0.63	0.548
Study design	2.82923	1.460152	(-0.0195, 0.0187)	1.94	0.094
Sample size	0.0009496	0.0003961	(0.00000386, 0.0018773)	2.37	0.049
Average age	0.002733	0.1090412	(-0.2551084, 0.2605744)	0.03	0.981
Publication year	0.1528608	0.1750213	(-0.2609988, 0.5667204)	0.87	0.411
Male ratio	11.30861	6.430022	(-3.895979, 26.51319)	1.76	0.122
Severity of carotid atherosclerosis lesions	3.274568	0.8557248	(1.2511, 5.298035)	3.83	0.006

### 3.7 Sensitivity analysis and publication bias

Given the pronounced heterogeneity in the association between bilirubin levels and carotid atherosclerosis, an additional sensitivity analysis was performed (**Supplementary Figure S3**). No significant alternations were detected when excluding one of the included studies and pooling the rest. Overall, this suggests that the primary results of this meta-analysis are relatively robust.

### 3.8 Publication bias

**Figure 5:**
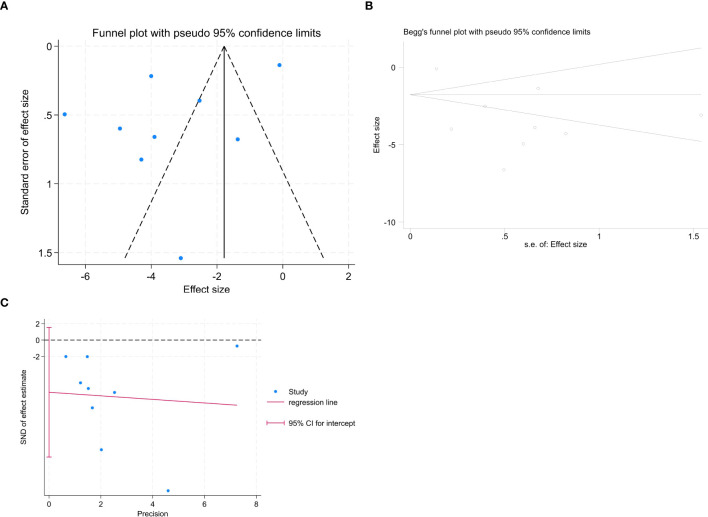
Publication bias analysis. **(A)** funnel plot; **(B)** Begg’s test; **(C)** Egger’s test.

To assess publication bias, a forest plot was drawn. The distribution on both sides of the graph appeared asymmetric (**Figure 5A**); however, further analyses using Begg’s (**Figure 5B**) and Egger’s (**Figure 5C**) tests indicated no significant publication bias among the articles included in this meta-analysis (Begg: z = 0.73, *P* = 0.466; Egger: *P*= 0.098, 95% CI [- 14.280, 1.536]).

## 4 Discussion

Carotid atherosclerosis is a main cause of ischemic stroke. Studies have found that nearly one-quarter of Chinese adults have increased CIMT or carotid plaque, which places a substantial burden on socio-economic and health care system (32). The current situation urges us to seek effective strategies for the management of carotid atherosclerosis. Previous studies have found that serum bilirubin and its metabolite HO-1 play a protective role against the progression of carotid atherosclerotic disease (33). However, whether serum bilirubin level is associated with carotid atherosclerosis remains controversial. Our results revealed that lower serum bilirubin levels might be associated with a higher risk of carotid atherosclerosis. What’s more, such an association was likewise significant in further subgroup analyses based on study design, geographic location, publication year, sample size, and average age. Sample size and severity of carotid atherosclerosis lesions(CIMT or carotid plaques) significantly affected the SMD of bilirubin levels in the univariate meta-regression.

To date, the underlying protective mechanism of serum bilirubin against carotid atherosclerosis remains unclear, but there are several possible explanations. Bilirubin, a natural endogenous antioxidant, scavenges reactive oxygen species (ROS) through its conjugated double bonds and active hydrogen atoms, mitigating oxidative stress damage to vascular endothelial cells and inhibiting atherosclerotic plaque formation (34, 35). It also activates the nuclear transcription factor erythroid 2-related factor 2 (Nrf2)/heme oxygenase (HO-1) signaling pathway, enhancing cellular antioxidant capacity and inhibiting apoptosis (36). HO-1 inhibits vascular smooth muscle cell (VSMC) proliferation and migration, reducing intimal accumulation and limiting plaque formation (37). Elevated plasma HO-1 levels are observed in patients with carotid plaques and are positively correlated with the severity of carotid atherosclerosis (38). Bilirubin exhibits anti-inflammatory effects by inhibiting macrophage polarization, complement cascade reactions, and the removal of circulating hemoglobin, protecting the vessel wall from inflammatory damage. It inhibits the NF-κB signaling pathway, reducing the production of pro-inflammatory cytokines such as TNF-α, IL-6, NLRP3, and NLRC4, thereby mitigating inflammatory responses in atherosclerotic lesions (36, 39, 40). Endothelial dysfunction, an early feature of atherosclerosis, is ameliorated by bilirubin through binding to lipids or albumin in atherosclerotic lesions and scavenging hydroxyl radicals and superoxide anions (41). Serum bilirubin levels are negatively correlated with the expression of E-selectin and intercellular adhesion molecule 1 (ICAM-1), which mediate leukocyte extravasation and monocyte recruitment in atherogenesis (12, 41). Animal studies indicate that mild hyperbilirubinemia prevents atherosclerosis progression by improving lipid profiles and modulating the mRNA expression of vascular cell adhesion molecule 1 (VCAM-1), ICAM-1, inducible nitric oxide synthase (iNOS), and lectin-like LDL receptor 1 (LOX-1) (42). Furthermore, bilirubin inhibits VSMC proliferation and migration, suppressing neoplastic intima proliferation and providing arterial wall protection, thus delaying atherosclerotic plaque formation (43). Recent studies show that bilirubin inhibits cholesterol biosynthesis by promoting HMGCR ubiquitination, reducing lipid accumulation in the arterial wall and improving atherosclerosis (44). Bvra deficiency, leading to bilirubin deficiency, enhances the proatherogenic phenotype, increasing atherogenesis risk and causing plaque destabilization (45). Changes in bilirubin levels play pivotal role in the development of carotid atherosclerosis.

Previous studies have proposed that serum bilirubin levels can serve as a predictor for the occurrence and progression of carotid atherosclerosis; however, the relationship between arteriosclerotic cardiovascular disease (ASCVD) and serum bilirubin levels has been inconsistent. Recently, some studies have reported that serum bilirubin is independently and inversely associated with the risks of carotid or femoral atherosclerosis (46, 47), while others have discovered a U-shaped dose-response relationship between bilirubin levels and the risk of coronary heart disease (CHD), particularly in men (48, 49). A Korean study involving patients with type 2 diabetes mellitus (T2DM) over an 8-year follow-up indicated that higher levels of total serum bilirubin were significantly associated with a lower risk of plaque progression in the carotid arteries (50). Furthermore, a meta-analysis encompassing 323,891 patients indicated that higher serum total bilirubin levels are an independent protective factor for ASCVD and are inversely associated with the prognosis of acute myocardial infarction (AMI), stroke, and peripheral arterial disease (PAD), yet positively correlated with in-hospital cardiovascular death and major adverse cardiac events (MACEs) (51).

In our study, serum bilirubin levels were negatively correlated with CIMT thickening ((SMD -4.77, 95% CI [-5.87, -3.67]; OR 0.68, 95% CI [0.55, 0.85])), but only weakly correlated carotid plaque (SMD -1.58, 95% CI [-3.21, 0.06]; OR 0.88, 95% CI [0.79, 0.99]). Subgroup analysis based on diagnostic criteria for carotid atherosclerosis found that serum bilirubin levels were significantly lower in patients with carotid atherosclerosis than in controls when CIMT ≥ 0.9 mm was used as a diagnostic criterion, whereas this was not observed when CIMT ≥ 1.2 mm or plaque formation, or CIMT ≥ 1.5 mm or focal thickening > 50%. A one-way meta-regression analysis revealed a significant relationship between the severity of carotid atherosclerosis lesions and serum total bilirubin levels (*P* < 0.05), which may account for the observed heterogeneity. CIMT thickening typically signifies the early and subclinical stages of carotid atherosclerosis, while the presence of carotid plaques and stenoses indicates more advanced stages of atherosclerotic progression (52). The formation of plaques involves irreversible and complex pathological processes, such as necrosis of lipid cores, rupture of the fibrous cap, and inflammatory cascades, which may reduce the protective effect of bilirubin. However, a cross-sectional study by Chun-Hua Jin et al. demonstrated that high-normal serum unconjugated bilirubin (UCB) is inversely associated with late carotid atherosclerotic lesions including carotid plaque and stenosis but not with CIMT, an early carotid atherosclerotic lesion (53). The primary distinction lies in the fact that CIMT is routinely measured in the distal 1 cm of the common carotid artery just before the bifurcation, whereas plaque formation and progression predominantly occur downstream in the internal carotid artery (54). In addition, different methods of measuring bilirubin, such as the bilirubin oxidase method and the vanadate oxidation method, may also be a source of heterogeneity.

Regarding study design, subgroup analyses showed that the combined effect in cross-sectional studies was consistent with the overall results, while it was not applicable in cohort studies. A total of 7 cross-sectional studies (24–30) and 2 cohort studies (23, 31) were included in our meta-analysis. Interestingly, we observed that the cross-sectional studies were all in subjects with hypertension, HIV-1 infection, obstructive sleep apnea, prediabetes, and hyperbilirubinemia, whereas the cohort studies were all based on healthy physical examination populations. Therefore, we speculate that the inconsistency may be due to differences in the number of cross-sectional and cohort studies, and the health status of the study populations.

Subgroup analyses of the study sample sizes revealed that serum total bilirubin levels in patients in the CAS group were significantly lower than those in the non-CAS group when the sample sizes were fewer than 200 individuals, exhibiting notably less heterogeneity. Similar conclusions were drawn for sample sizes of 200 or more, yielding heterogeneity did not change significantly. One-way meta-regression analysis showed a significant effect of sample size on serum total bilirubin levels (*P*<0.05), which may contribute to the observed heterogeneity. For instance, this study involved individuals from three different countries, with the smallest sample size being 80 and the largest reaching 4,360. It is necessary to conduct large-scale, long-term follow-up cohort studies to validate our findings.

To our knowledge, this study is the first systematic review and meta-analysis investigating the correlation between serum bilirubin levels and carotid atherosclerosis. We employed standardized methods for literature identification and assessment, conducted a comprehensive search, and ensured rigorous data processing and quality assessment. Subgroup analyses and meta-regression indicated that sample size severity of carotid atherosclerosis might be the source of heterogeneity. The results suggest that higher serum bilirubin levels could act as a protective factor against carotid atherosclerosis, which may prompt future research scholars to increase attention on this topic. Importantly, our study offers new insights into the clinical application of serum bilirubin levels as a biomarker for the early diagnosis of carotid atherosclerosis or as an adjunct to therapy.

However, our meta-analysis has several limitations. First, the included studies, primarily observational, indicate a potential association between serum bilirubin and carotid atherosclerosis, but could not be used to diagnose carotid atherosclerosis or establish causality. Second, our meta-analysis included only studies published in English, predominantly from Europe and Asia, specifically involving populations from China, Turkey, and Italy. Some of the studies had small sample sizes, which may limit the representativeness of the results. Caution is therefore warranted in interpreting these findings. Additionally, the variables adjusted for varied across studies, potentially introducing bias in the estimation of the pooled SMD and OR. Fourth, the criteria for defining carotid atherosclerosis differed among the original studies, with varying thresholds affecting the frequency of exposure. Finally, results based on observational studies cannot demonstrate a causal relationship between serum total bilirubin levels and carotid atherosclerosis. Consequently, there is a pressing need for higher-quality, large-scale, prospective studies with long-term follow-ups, as well as foundational research, to yield more reliable evidence.

## 5 Conclusion

The results of the meta-analysis revealed that serum bilirubin levels were significantly lower in patients with carotid atherosclerosis compared to controls, revealing a significant relationship between serum bilirubin levels and the risk of carotid atherosclerosis. However, the magnitude of this association may depend on the stage of carotid atherosclerosis development. By examining the relationship between bilirubin and carotid atherosclerosis, this meta-analysis establishes a basis for the development of more convenient diagnostic biomarkers and effective therapeutic targets. Further prospective cohort studies and randomized controlled trials are necessary to validate our findings.
