# Supplementary material for: Association between serum bilirubin levels and carotid atherosclerosis: a systematic review and meta-analysis
Source: Front Endocrinol (Lausanne). 2025 Jun 19;16:1451465. doi: 10.3389/fendo.2025.1451465 (PMC12221923; doi:10.3389/fendo.2025.1451465)
Supplement: Supplementary file 1 [file DataSheet1.docx]

Supplementary Material

**Supplementary Tables**

**Supplementary Table S1.** Specific description of the search strategy

| **Database** | **#** | **Search strategy** | **Results** |
| --- | --- | --- | --- |
| PubMed | 1 | "Bilirubin"[Mesh] | 26,672 |
|  | 2 | ((((((((((((((((((Bilirubin) OR (Bilirubin IX alpha)) OR (Bilirubin, (4E)-Isomer)) OR (Bilirubin, (4E,15E)-Isomer)) OR (Hematoidin)) OR (Bilirubin, Disodium Salt)) OR (Disodium Salt Bilirubin)) OR (Bilirubin, Monosodium Salt)) OR (Monosodium Salt Bilirubin)) OR (delta-Bilirubin)) OR (delta Bilirubin)) OR (Bilirubin, (15E)-Isomer)) OR (Bilirubin, Calcium Salt)) OR (Calcium Salt Bilirubin)) OR (Salt Bilirubin, Calcium)) OR (Calcium Bilirubinate)) OR (Bilirubinate, Calcium)) OR (bilirubinaemia)) OR (hyperbilirubinemia) | 78,337 |
|  | 3 | #1 OR #2 | 78,337 |
|  | 4 | ("Carotid Artery Diseases"[Mesh]) OR ("Carotid Intima-Media Thickness"[Mesh]) | 57,690 |
|  | 5 | (((((((((((((((((((((((((((((((((Artery Disease, Carotid) OR (Artery Diseases, Carotid)) OR (Carotid Artery Disease)) OR (Carotid Artery Disorders)) OR (Artery Disorder, Carotid)) OR (Artery Disorders, Carotid)) OR (Carotid Artery Disorder)) OR (Disorders, Carotid Artery)) OR (Arterial Diseases, Carotid)) OR (Arterial Disease, Carotid)) OR (Carotid Arterial Disease)) OR (Carotid Arterial Diseases)) OR (Carotid Atherosclerosis)) OR (Carotid Atheroscleroses)) OR (Carotid Atherosclerotic Disease)) OR (Atherosclerotic Disease, Carotid)) OR (Atherosclerotic Diseases, Carotid)) OR (Carotid Atherosclerotic Diseases)) OR (Internal Carotid Artery Diseases)) OR (Arterial Diseases, Internal Carotid)) OR (Internal Carotid Artery Disease)) OR (Arterial Diseases, Common Carotid)) OR (Common Carotid Artery Diseases)) OR (Common Carotid Artery Disease)) OR (External Carotid Artery Diseases)) OR (Arterial Diseases, External Carotid)) OR (Carotid Intima Media Thickness)) OR (Intima-Media Thickness, Carotid)) OR (Carotid Thickening)) OR (Carotid Atherosclerotic Plaque)) OR (Carotid Plaque)) OR (Carotid Artery Plaque)) OR (Carotid Arterial Plaque)) OR (Carotid Stenosis) | 94,501 |
|  | 6 | #4 OR #5 | 94,501 |
|  | 7 | #3 AND #6 | 81 |
| Web of science | 1 | TS=(Bilirubin or Bilirubin IX alpha or Bilirubin, (4E)-Isomer or Bilirubin, (4E,15E)-Isomer or Hematoidin or Bilirubin, Disodium Salt or Disodium Salt Bilirubin or Bilirubin, Monosodium Salt or Monosodium Salt Bilirubin or delta-Bilirubin or delta Bilirubin or Bilirubin, (15E)-Isomer or Bilirubin, Calcium Salt or Calcium Salt Bilirubin or Salt Bilirubin, Calcium or Calcium Bilirubinate or Bilirubinate, Calcium or bilirubinaemia or hyperbilirubinemia) | 105,017 |
|  | 2 | TS=(Artery Disease, Carotid OR Artery Diseases, Carotid OR Carotid Artery Disease OR Carotid Artery Disorders OR Artery Disorder, Carotid OR Artery Disorders, Carotid OR Carotid Artery Disorder OR Disorders, Carotid Artery OR Arterial Diseases, Carotid OR Arterial Disease, Carotid OR Carotid Arterial Disease OR Carotid Arterial Diseases OR Carotid Atherosclerosis OR Carotid atherosclerosis OR Carotid Atherosclerotic Disease OR Atherosclerotic Disease, Carotid OR Atherosclerotic Diseases, Carotid OR Carotid Atherosclerotic Diseases OR Internal Carotid Artery Diseases OR Arterial Diseases, Internal Carotid OR Internal Carotid Artery Disease OR Arterial Diseases, Common Carotid OR Common Carotid Artery Diseases OR Common Carotid Artery Disease OR External Carotid Artery Diseases OR Arterial Diseases, External Carotid OR Carotid Intima-Media Thickness OR Intima-Media Thickness, Carotid OR Carotid Thickening OR Carotid Atherosclerotic Plaque OR Carotid Plaque OR Carotid Artery Plaque OR Carotid Arterial Plaque OR Carotid Stenosis) | 153,123 |
|  | 3 | #1 AND #2 | [17](https://webofscience.clarivate.cn/wos/alldb/summary/354aa0ce-0908-4ac4-a20c-c899ae5b3fad-b5acef40/relevance/1)2 |
| Embase | 1 | 'bilirubin'/exp | 119,781 |
|  | 2 | 'Bilirubin':ab,ti OR 'Bilirubin IX alpha':ab,ti OR 'Bilirubin, (4E)-Isomer':ab,ti OR 'Bilirubin, (4E,15E)-Isomer':ab,ti OR 'haematoidin':ab,ti OR 'Bilirubin, Disodium Salt':ab,ti OR 'Disodium Salt Bilirubin':ab,ti OR 'Bilirubin, Monosodium Salt':ab,ti OR 'Monosodium Salt Bilirubin':ab,ti OR 'delta-Bilirubin':ab,ti OR 'delta Bilirubin':ab,ti OR 'Bilirubin, (15E)-Isomer':ab,ti OR 'Bilirubin, Calcium Salt':ab,ti OR 'Calcium Salt Bilirubin':ab,ti OR 'Salt Bilirubin, Calcium':ab,ti OR 'Calcium Bilirubinate':ab,ti OR 'Bilirubinate, Calcium':ab,ti OR 'bilirubinemia':ab,ti OR 'hyperbilirubinemia':ab,ti | 86,832 |
|  | 3 | #1 OR #2 | 144,885 |
|  | 4 | 'carotid atherosclerosis'/exp | 11,995 |
|  | 5 | 'carotid intima-media thickness'/exp | 3,551 |
|  | 6 | 'carotid artery diseases':ab,ti OR 'artery disease, carotid':ab,ti OR 'artery diseases, carotid':ab,ti OR 'carotid artery disease':ab,ti OR 'carotid artery disorders':ab,ti OR 'artery disorder, carotid':ab,ti OR 'artery disorders, carotid':ab,ti OR 'carotid artery disorder':ab,ti OR 'disorders, carotid artery':ab,ti OR 'arterial diseases, carotid':ab,ti OR 'arterial disease, carotid':ab,ti OR 'carotid arterial disease':ab,ti OR 'carotid arterial diseases':ab,ti OR 'carotid atheroscleroses':ab,ti OR 'carotid atherosclerotic disease':ab,ti OR 'atherosclerotic disease, carotid':ab,ti OR 'atherosclerotic diseases, carotid':ab,ti OR 'carotid atherosclerotic diseases':ab,ti OR 'internal carotid artery diseases':ab,ti OR 'arterial diseases, internal carotid':ab,ti OR 'internal carotid artery disease':ab,ti OR 'arterial diseases, common carotid':ab,ti OR 'common carotid artery diseases':ab,ti OR 'common carotid artery disease':ab,ti OR 'external carotid artery diseases':ab,ti OR 'arterial diseases, external carotid':ab,ti OR 'carotid intima media thickness':ab,ti OR 'intima-media thickness, carotid':ab,ti OR 'carotid thickening':ab,ti OR 'carotid atherosclerotic plaque':ab,ti OR 'carotid plaque':ab,ti OR 'carotid artery plaque':ab,ti OR 'carotid arterial plaque':ab,ti OR 'carotid stenosis':ab,ti | 32,308 |
|  | 7 | #4 OR #5 OR #6 | 39,592 |
|  | 8 | #3 AND #7 | 127 |

**Supplementary Table S2.** Risk of bias assessment (Newcastle-Ottawa Quality Assessment Scale criteria).

| Study | Selection | | | | Comparability | Outcome | | | Quality  score |
| --- | --- | --- | --- | --- | --- | --- | --- | --- | --- |
|  | Is the case definition adequate/ Representativeness of the exposed cohort | Representativeness of the cases/ Selection of the non-exposed cohort | Selection of Controls/ Ascertainment of exposure to implants | Definition of Controls/ Demonstration that outcome of interest was not present at start of study | Comparability of cases and controls on the basis of the design or analysis/ Comparability of cohorts on the basis of the design or analysis | Ascertainment of exposure/ Assessment of outcome | Same method of ascertainment for cases and controls/ Was follow up long enough for outcomes to occur (>5 years)? | Non-Response Rate/ Adequacy of follow up of cohorts (>5 years). |  |
| Zhao J(2019） | * | * | * | * | * | * | * | * | 8 |
| Tang L H(2019） | * | * | * | * | * | * | * | * | 8 |
| Muccini C(2018) | * | * | * | * | ** | * | * | * | 9 |
| Lyu Q S(2018) | * | * | * | * | * | * | * | * | 8 |
| Duman H(2018) | * | * | * | * | * | * | * | * | 8 |
| Hamur H(2016) | * | * | * | * | * | * | * | * | 8 |
| Jiang S M(2012) | * | * | * | * | * | * | * | * | 8 |
| Yang X F(2009) | * | * | * | * | ** | * | * | * | 9 |
| Yang X F(2023) | * | * | * | * | ** | * | 无 | * | 8 |

**Supplementary Table S3.**

GRADE evidence table for the association between serum bilirubin level and the risk of carotid atherosclerosis.

| **Certainty assessment** | | | | | | | **№ of patients** | | **Effect** | | **Certainty** | **Importance** |
| --- | --- | --- | --- | --- | --- | --- | --- | --- | --- | --- | --- | --- |
| **№ of studies** | **Study design** | **Risk of bias** | **Inconsistency** | **Indirectness** | **Imprecision** | **Other considerations** | **[Participants]** | **[Cases(%)]** | **Relative (95% CI)** | **Absolute (95% CI)** |  |  |
| **Increased carotid intima-media thickness (assessed with: carotid arery ultrasound)** | | | | | | | | | | | | |
| 5 | non-randomised studies | serious^a^ | not serious^b^ | not serious | not serious | all plausible residual confounding would reduce the demonstrated effect | 396/1362 | 29.1% | **OR 0.68** (0.55 to 0.85) | **7 fewer per 100** (from 11 fewer to 3 fewer) | ⨁⨁◯◯ Low^a,b^ |  |
| **Carotid plaque (assessed with: carotid artery ultrasound)** | | | | | | | | | | | | |
| 4 | non-randomised studies | serious^c^ | serious^d^ | not serious | not serious | all plausible residual confounding would reduce the demonstrated effect dose response gradient | 2937/5661 | 51.9% | **OR 0.88** (0.79 to 0.99) | **3 fewer per 100** (from 6 fewer to 0 fewer) | ⨁⨁◯◯ Low^c,d^ |  |

**CI:** confidence interval; **OR:** odds ratio

1. **Explanations**

a. Downgraded once since participants in all 5 studies had comorbidities： in 1 study participants had essential hypertension; in 2 studies they were all prehypertensive; in 1 study they were all OSA patients, of whom approximately 56% had hypertension; and in 1 study they were pre-diabetic. However, four of the studies adequately adjusted for the potential confounding effects of these comorbidities, such as blood pressure.

b. I^2^= 70.5%, the results of all 5 studies were in the same direction (inverse relationship). Subgroup analysis explained 2 studies with study countries in Turkey, Italy, with significantly lower heterogeneity. Therefore no downgrading.

c. Downgraded once since two of the 4 studies were highly weighted (82.4%) and were in healthy physical examined populations with a low risk of selection bias, one of which was adequately adjusted before grouping using propensity score matching for Age, sex, BMI, smoking, hypertension, diabetes, blood lipids, liver, and kidney function indexes,; 2 had participants with comorbidities, one with HIV infection; and one with essential hypertension, of which approximately 51% had diabetes.

d. Downgraded once, I^2^ = 71.3%

## Supplementary Figures

**Supplementary Figure S1.** Subgroup analysis of meta-analysis (Random-Effects Model, SMD). (A) Country (B) Study design. (C) Sample size. (D) Average age. (E) Publication year. (F) Diagnostic criteria for CAS


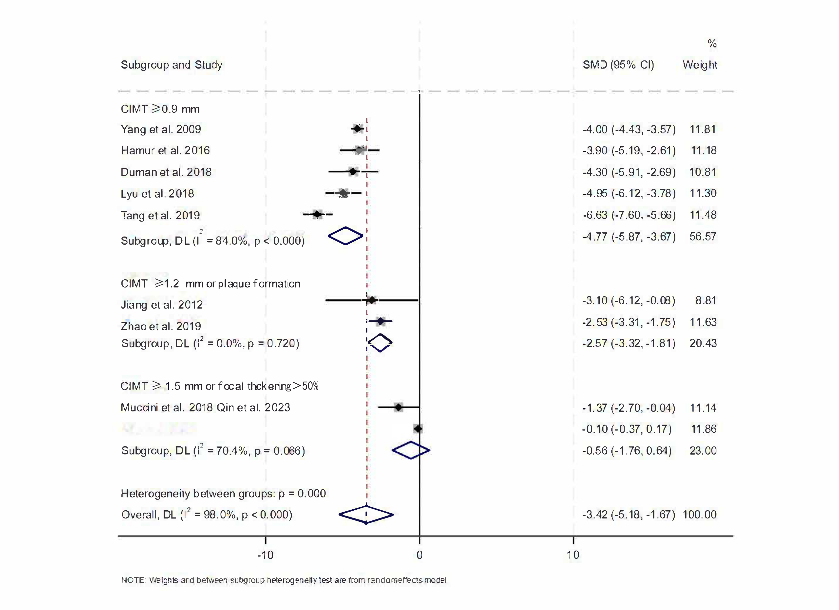


A

D

C

B

E

F

**Supplementary Figure S2.** Meta-regression analysis. (A) Country. (B) Study design. (C) Sample size. (D) Average age. (E) Publication year. (F) Male ratio. (G) Severity of carotid atherosclerosis lesions.


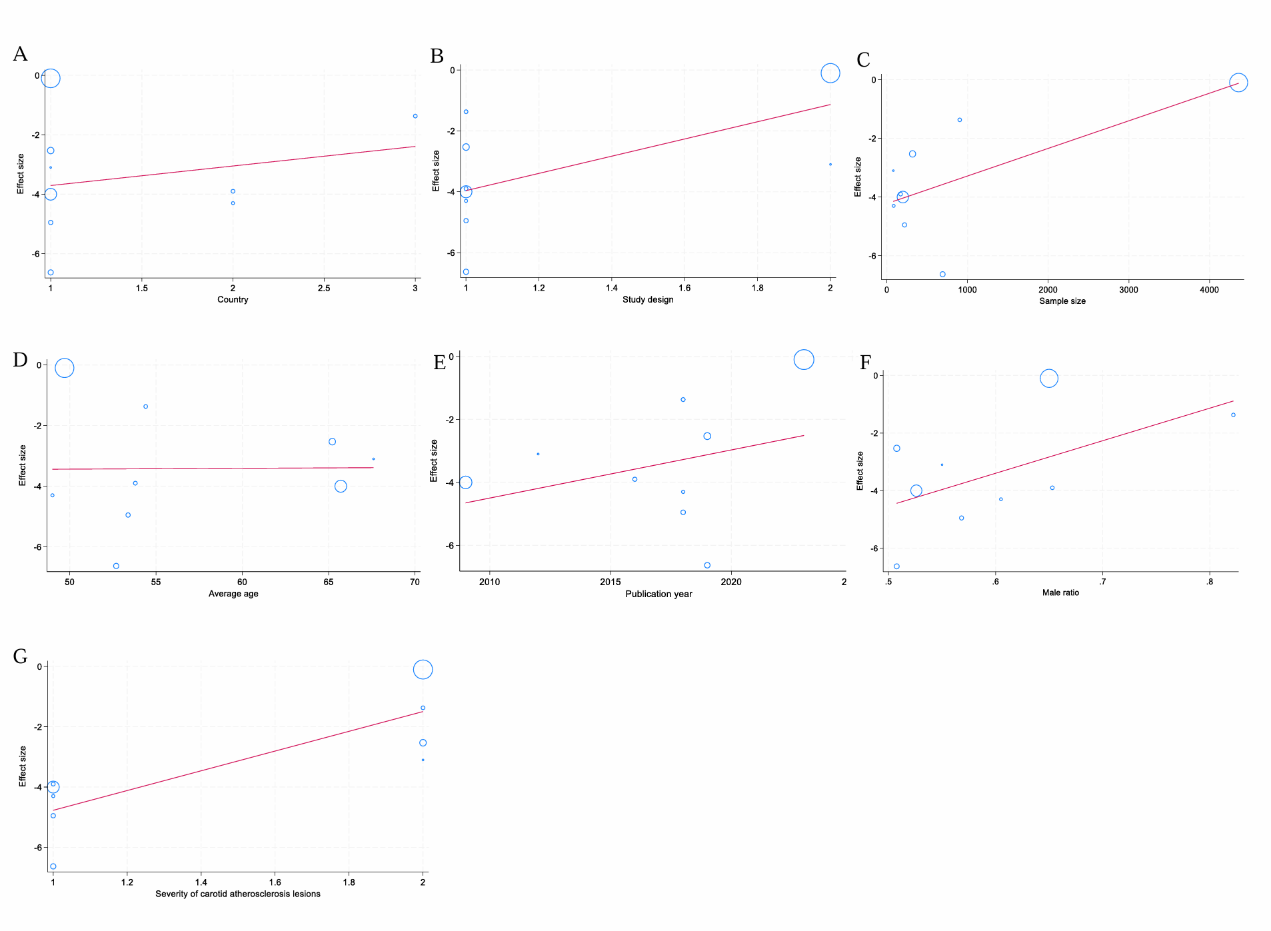

**Supplementary Figure S3.** Sensitivity analysis of the pooled standard mean variance (SMD).
